# Interpretable machine learning model for early prediction of deep vein thrombosis in elderly ICU patients: multicenter development and external validation

**DOI:** 10.1186/s12911-026-03747-z

**Published:** 2026-08-08

**Authors:** Jiabao Yang, Xiangrong Zhao, Juan Ren

**Affiliations:** 1 Department of Radiotherapy, The First Affiliated Hospital of Xi’an Jiaotong University, Xi’an, China; 2 Center of PRaG Therapy, The Second Affiliated Hospital of Soochow University, Suzhou, China

**Keywords:** Deep vein thrombosis, Intensive care unit, Machine learning, Clinical decision support, Risk prediction, Explainable artificial intelligence

## Abstract

**Background:**

Deep vein thrombosis (DVT) is a frequent complication in elderly intensive care unit (ICU) patients, while reliable prediction tools for this population remain limited. This study aimed to develop and externally validate an interpretable machine learning model for early prediction of DVT in critically ill elderly patients.

**Methods:**

In this multicenter retrospective study, ICU patients aged ≥ 75 years from two tertiary medical centers in China were enrolled. Patients with pre-existing DVT were excluded. A development cohort (Suzhou, *n* = 1,717) and an external validation cohort (Xi’an, *n* = 533) were established. Clinical variables collected within 24 h after ICU admission were used to predict incident DVT during ICU stay. Least absolute shrinkage and selection operator regression was applied for feature selection, and four machine learning models were developed and compared. Model discrimination, calibration, and interpretability were evaluated using area under the curve (AUC), calibration analysis, and SHapley Additive exPlanations (SHAP).

**Results:**

DVT occurred in 15.2% and 21.0% of patients in the development and external validation cohorts, respectively. Among the evaluated models, XGBoost achieved the best performance, with AUCs of 0.843 in internal validation and 0.811 in external validation. Calibration analysis demonstrated good agreement between predicted and observed risks. Key predictors included D-dimer, C-reactive protein, platelet-to-lymphocyte ratio, procalcitonin, mechanical ventilation, and mechanical thromboprophylaxis. SHAP analysis quantified the contribution of individual predictors.

**Conclusions:**

We developed and externally validated an interpretable machine learning model for early DVT prediction in elderly ICU patients. The model may support risk stratification and clinical decision-making in critical care settings.

**Supplementary Information:**

The online version contains supplementary material available at https://doi.org/10.1186/s12911-026-03747-z.

## Introduction

Deep vein thrombosis (DVT) remains a major complication in critically ill patients and a leading cause of preventable morbidity and mortality in the intensive care unit (ICU) among elderly populations [1]. The burden of venous thromboembolism is particularly high in older adults due to reduced mobility, physiological decline, and multiple comorbidities [2, 3]. Despite routine thromboprophylaxis, the incidence of DVT in ICU patients remains substantial, highlighting the need for more accurate early risk stratification strategies [4, 5].

Conventional risk assessment models, such as the Caprini and Padua scores, were developed for general medical and surgical populations and have shown limited predictive performance in critically ill patients [6, 7]. Their inadequate adaptability to ICU-specific pathophysiological conditions may lead to under- or overuse of anticoagulation, increasing the risk of either thrombosis or bleeding [8, 9]. Therefore, there is an unmet need for more individualized and data-driven prediction tools to support clinical decision-making in this population [10].

Recent advances in machine learning (ML) have enabled the development of predictive models capable of capturing complex nonlinear relationships among clinical variables [11, 12]. Methods such as logistic regression, random forest, support vector machine, and eXtreme Gradient Boosting have been widely applied in critical care research with promising performance [13, 14]. In parallel, interpretable ML approaches, including SHapley Additive exPlanations (SHAP), have improved model transparency and clinical interpretability, facilitating potential integration into clinical decision support systems [15–17].

Emerging evidence suggests that immune-inflammatory processes may contribute to thrombus formation through mechanisms of immunothrombosis [18, 19]. However, the incremental value of immune-inflammatory biomarkers for DVT risk prediction in elderly ICU patients remains unclear [20–22].

Patients aged ≥ 75 years were specifically selected because this age threshold is commonly used to define the “oldest-old” population, who exhibit substantially higher risks of frailty, multimorbidity, thromboembolic events, and adverse ICU outcomes than younger older adults. Existing DVT prediction models have rarely focused specifically on this particularly vulnerable population. In this multicenter retrospective study, we developed and externally validated interpretable machine learning models for early prediction of DVT in ICU patients aged ≥ 75 years using routinely available clinical variables. We further evaluated model generalizability in an independent external cohort and explored the potential incremental value of immune-inflammatory biomarkers. Our aim was to develop a clinically applicable and interpretable tool to support early risk stratification and decision-making for thromboprophylaxis in critically ill elderly patients.

## Methods

### Study design and population

This multicenter retrospective study included critically ill elderly patients admitted to the intensive care units (ICUs) of two tertiary medical centers in China: The Second Affiliated Hospital of Soochow University and The First Affiliated Hospital of Xi’an Jiaotong University.

**Fig. 1:**
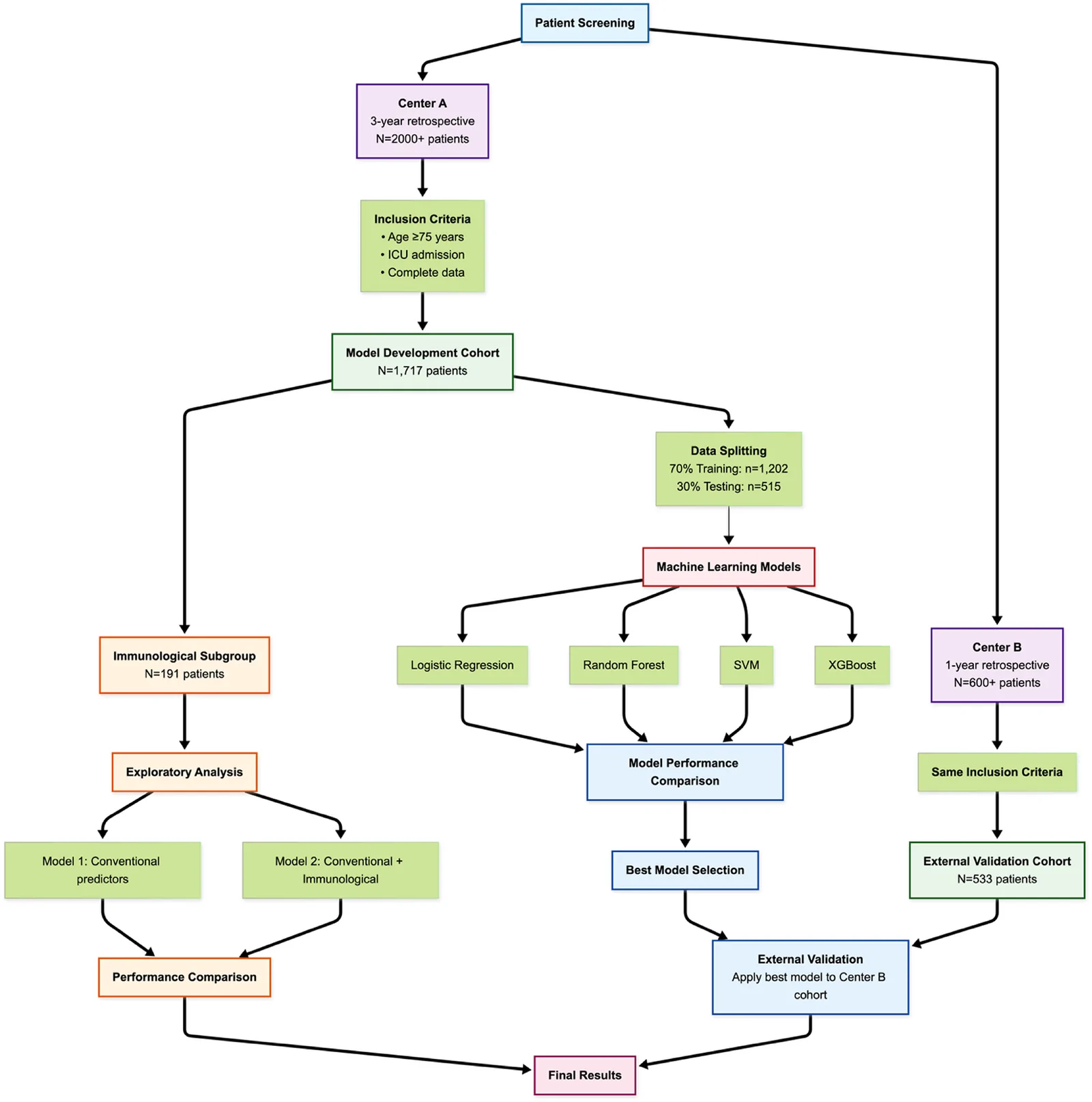
Flowchart of patient selection and study design. After application of the exclusion criteria, 27 patients with pre-existing DVT were excluded from the Suzhou cohort and 11 from the Xi’an cohort. The final development cohort included 1,717 elderly ICU patients from The Second Affiliated Hospital of Soochow University and was randomly divided into training (70%, *n* = 1202) and internal test (30%, *n* = 515) sets. An exploratory immunological subgroup (*n* = 191) with available immune biomarker data was additionally analyzed. External validation was performed using an independent cohort of 533 patients from The First Affiliated Hospital of Xi’an Jiaotong University

The development cohort included patients admitted to the ICU of The Second Affiliated Hospital of Soochow University between January 1, 2022 and December 31, 2024. A total of 1,717 eligible patients aged ≥ 75 years were included. Among them, 191 patients with available immune-inflammatory biomarker data were included in an exploratory subgroup analysis. An independent external validation cohort was obtained from the ICU of The First Affiliated Hospital of Xi’an Jiaotong University between January 1, 2024 and December 31, 2024, including 533 patients who met the same eligibility criteria (Fig. 1). An age threshold of ≥ 75 years was adopted because this population represents the oldest-old group, which is characterized by a particularly high burden of frailty, multimorbidity, and thromboembolic risk. Focusing on this homogeneous high-risk population was intended to improve the clinical applicability of the prediction model for elderly ICU patients.

The study was conducted in accordance with the Declaration of Helsinki and approved by the institutional ethics committees of both participating centers. The requirement for informed consent was waived due to the retrospective nature of the study.

### Inclusion and exclusion criteria

Patients were included if they met the following criteria: (1) age ≥ 75 years; (2) ICU stay ≥ 48 h; (3) at least one lower-extremity venous Doppler ultrasound examination during hospitalization; (4) availability of essential clinical and laboratory data; and (5) complete documentation of thromboprophylaxis and mechanical ventilation status.

Patients were excluded if they had: (1) pre-existing DVT at ICU admission; (2) known coagulation disorders or active bleeding; (3) therapeutic anticoagulation at ICU admission; (4) expected survival < 48 h; or (5) missing key predictor variables.

### Data collection and predictor variables

All predictor variables were extracted from electronic medical records and collected within the first 24 h after ICU admission. To ensure inter-center consistency, only routinely available variables measured in both institutions were included. Variables with inter-center missingness > 15% were excluded.

Demographic characteristics, comorbidities, physiological parameters, and laboratory measurements were collected at ICU admission. For laboratory variables with temporal variability, including D-dimer and inflammatory markers, the earliest available measurement obtained within the first 24 h after ICU admission was used. To avoid information leakage, only laboratory measurements obtained before the diagnosis of DVT were included in model development. Measurements obtained after DVT diagnosis were excluded. The final dataset included 17 routinely available clinical variables: age, sex, body mass index (BMI), APACHE II score, diabetes, hypertension, malignancy, pharmacological thromboprophylaxis, mechanical thromboprophylaxis, mechanical ventilation, orthopedic surgery, D-dimer, procalcitonin (PCT), C-reactive protein (CRP), white blood cell count (WBC), platelet-to-lymphocyte ratio (PLR), and neutrophil-to-lymphocyte ratio (NLR). In the exploratory subgroup, additional immune-inflammatory biomarkers were analyzed, including T-lymphocyte subsets, natural killer cells, B lymphocytes, and circulating cytokines.

Missing continuous variables were imputed using multiple imputation by chained equations (MICE), whereas missing categorical variables were imputed using mode imputation. The proportions of missing data for all candidate predictors before imputation are summarized in Supplementary Table S1.

### Outcome definition

The primary outcome was new-onset lower-extremity deep vein thrombosis (DVT) occurring during ICU stay. DVT was diagnosed using standardized Doppler ultrasonography protocols and confirmed by radiology reports. Patients with DVT identified within 24 h before or after ICU admission were considered to have pre-existing DVT and were excluded. Both symptomatic and screening-detected DVT events were included. All outcomes were independently verified through review of radiology and medical records. Predictor variables were collected before DVT diagnosis to ensure temporal validity and prevent information leakage.

### Model development and validation

The development cohort was randomly divided into a training set (70%) and an internal test set (30%). Feature selection was performed using least absolute shrinkage and selection operator (LASSO) regression. LASSO was selected because it simultaneously performs variable selection and coefficient regularization, thereby reducing model complexity and the risk of overfitting in datasets with multiple potentially correlated predictors. Compared with traditional stepwise selection methods based on information criteria (e.g., AIC or BIC), LASSO generally provides more stable feature selection and has been widely adopted in predictive modeling and machine learning studies. Feature selection and model development were performed exclusively within the development cohort, while the independent external validation cohort was used solely for model evaluation, thereby minimizing the risk of data leakage. Logistic regression was used as a conventional baseline model for comparison with machine learning approaches.

For the baseline logistic regression (LR) models, the events-per-variable (EPV) ratio was calculated to assess model stability, with an EPV ≥ 10 considered acceptable. Multicollinearity among predictors was evaluated using the variance inflation factor (VIF), and a VIF value < 5 was considered indicative of the absence of substantial multicollinearity. Detailed EPV calculations and VIF results are presented in Supplementary Tables S2 and S3.

As a sensitivity analysis, all machine learning models were additionally developed using the complete set of candidate clinical predictors without LASSO-based feature selection, and their predictive performance was compared with that of the primary LASSO-based models.

Multiple machine learning algorithms, including logistic regression, random forest, support vector machine, and extreme gradient boosting (XGBoost), were developed and compared. Model training incorporated 10-fold cross-validation within the training dataset. Model performance was evaluated in both internal and external cohorts using discrimination and calibration metrics. Discrimination was assessed using area under the receiver operating characteristic curve (AUC), sensitivity, specificity, accuracy, and F1-score. Calibration was evaluated using calibration plots and Brier score. Bootstrap resampling (1,000 iterations) was used to estimate 95% confidence intervals for performance metrics. For all machine learning algorithms, predicted probabilities were converted into binary classifications using a fixed probability threshold of 0.5. The same threshold was applied consistently in the internal test cohort and the external validation cohort when calculating sensitivity, specificity, accuracy, F1-score, and confusion matrices.

### Sensitivity analysis

To evaluate the dependence of model performance on D-dimer, an additional sensitivity analysis was performed. D-dimer was excluded from the candidate predictor set, after which LASSO feature selection was repeated using the remaining clinical variables. All four machine learning models (logistic regression, random forest, support vector machine, and XGBoost) were subsequently retrained using the same data partition, model development strategy, and evaluation procedures as in the primary analysis.

### Model calibration

Calibration was primarily evaluated using graphical calibration plots together with calibration intercepts and slopes. An intercept of 0 and a slope of 1 indicated perfect calibration.

### Exploratory immunological subgroup analysis

An exploratory analysis was conducted in patients with available immune-inflammatory biomarker data (*n* = 191) to evaluate potential incremental predictive value. LASSO regression using the lambda.1se criterion was applied for feature selection. As a sensitivity analysis, feature selection was repeated using the alternative lambda.min criterion to evaluate the robustness of biomarker selection. Two logistic regression models were constructed: a baseline model using clinical predictors and an extended model incorporating selected immune-inflammatory biomarkers. Model performance was assessed using discrimination metrics with bootstrap resampling (1,000 iterations). This analysis was exploratory in nature and was not intended for direct comparison with the primary predictive models.

### Model interpretability

Model interpretability was assessed using SHapley Additive exPlanations (SHAP), which quantified the contribution of each feature to model predictions at both global and individual levels, enabling transparent interpretation of model outputs.

### Clinical application tool

To facilitate clinical use, a web-based risk calculator was developed based on the final XGBoost model to enable individualized DVT risk estimation using routinely available clinical variables.

### Statistical analysis

All analyses were performed using R software (version 4.5.0). Continuous variables were assessed for normality using the Shapiro–Wilk test in combination with visual inspection of histograms and Q–Q plots. Normally distributed variables were summarized as mean ± standard deviation (SD), whereas non-normally distributed variables were presented as median (interquartile range [IQR]). Categorical variables were expressed as frequencies and percentages. Comparisons of continuous variables between cohorts were performed using one-way analysis of variance (ANOVA) for approximately normally distributed variables and the Kruskal–Wallis test for non-normally distributed variables, whereas categorical variables were compared using the chi-square test, as appropriate. Because baseline comparisons were descriptive rather than inferential, standardized mean differences (SMDs) were used to describe cohort comparability rather than significance testing, with SMD < 0.10 indicating negligible imbalance. All statistical tests were two-sided, and *p* < 0.05 was considered statistically significant.

## Results

### Patient characteristics

The study flow is shown in Fig. 1. After application of exclusion criteria, 27 patients with pre-existing DVT in the Suzhou cohort and 11 in the Xi’an cohort were excluded. The final study population included the Suzhou development cohort (*n* = 1,717), an exploratory immunological subgroup (*n* = 191), and the Xi’an external validation cohort (*n* = 533).

The incidence of DVT was 15.2% in the Suzhou cohort and 21.0% in the Xi’an cohort. Baseline characteristics are summarized in Table 1. The immunological subgroup generally exhibited a similar distribution of baseline characteristics to the overall Suzhou cohort, suggesting that patients with available immune biomarker measurements were broadly representative of the development cohort. Because the immunological subgroup was nested within the full Suzhou cohort, this comparison is intended to describe cohort characteristics rather than to provide formal statistical inference. Compared with the Suzhou cohort, the Xi’an validation cohort showed small-to-moderate differences in age, diabetes, hypertension, and D-dimer levels (SMD 0.1–0.2), while other variables demonstrated minimal imbalance (SMD < 0.1).

**Table 1:** Baseline characteristics of the development and validation cohorts

Characteristics	Suzhou Full Cohort (*n* = 1717)	Suzhou Immune Subgroup (*n* = 191)	SMD (Suzhou Full vs. Immune)	Xi’an Validation Cohort (*n* = 533)	SMD (Suzhou Full vs. Xi’an)
Age, years					
Mean ± SD	82.01 ± 5.96	82.86 ± 6.11	0.142	81.12 ± 6.23	0.146
75–84	1283(74.4%)	132(69.1%)		411(77.1%)	
≥ 85	434(25.3%)	59(30.9%)		122(22.9%)	
Sex			0.039		0.013
Male	707(41.2%)	75 (39.3%)		216(40.5%)	
Female	1010(58.8%)	116(60.7%)		317(59.5%)	
BMI, kg/m^2^	19.89 ± 2.25	19.97 ± 2.45	0.034	19.82 ± 2.42	0.032
< 24	1616(94.1%)	176(92.1%)		493(92.5%)	
≥ 24	101(5.9%)	15(7.9%)		40(7.5%)	
APACHE II	18.96 ± 6.28	19.26 ± 6.49	0.047	19.65 ± 6.55	0.108
Comorbidities					
Diabetes	1047 (61.0%)	120(62.8%)	0.038	360(67.5%)	0.137
Hypertension	1174 (68.4%)	135 (70.7%)	0.050	410(76.9%)	0.193
Malignancy	359 (20.9%)	39(20.4%)	0.012	136(25.5%)	0.109
Treatments					
Pharmacological thromboprophylaxis	671(39.1%)	70 (36.6%)	0.050	189(35.5%)	0.075
Mechanical thromboprophylaxis	719 (41.9%)	85(44.5%)	0.053	193(36.2%)	0.116
Orthopedic surgery	219(12.8%)	31 (16.2%)	0.099	89(16.7%)	0.111
Mechanical ventilation	511(29.8%)	66 (34.6%)	0.103	182(34.1%)	0.094
Hematology examination					
D-dimer, mg/L	0.73(0.42–1.18)	0.69(0.40–1.10)	0.074	0.81(0.47–1.36)	0.126
NLR	2.61(1.65–5.07)	2.54(1.59–4.82)	0.060	2.79(1.71–5.24)	0.034
PLR	118(84–191)	114(79–178)	0.093	121(86–194)	0.015
WBC, ×10^9^/L	7.45 ± 3.35	7.36 ± 4.82	0.022	7.52 ± 3.62	0.018
PCT, ng/mL	0.29(0.12–0.70)	0.33(0.14–0.75)	0.066	0.35(0.15–0.83)	0.089
CRP, mg/L	10.8(7.2–15.6)	11.3(7.5–16.1)	0.048	11.1(7.4–16.0)	0.057
Outcome					
DVT	261(15.2%)	36(18.8%)	0.097	112(21%)	0.151

Continuous variables are presented as mean ± standard deviation (SD) for approximately normally distributed variables and median (interquartile range [IQR]) for non-normally distributed variables. Categorical variables are presented as number (percentage). Group comparisons were performed using analysis of variance, Kruskal–Wallis, or chi-square tests, as appropriate. Standardized mean differences (SMDs) were calculated to assess between-group balance, with SMD < 0.10 indicating minimal imbalance

### Feature selection using LASSO regression

Feature selection was performed using LASSO (Least Absolute Shrinkage and Selection Operator) regression with a stratified selection strategy. Distinct λ (lambda) criteria were applied according to variable types. For conventional clinical variables, the lambda.min criterion was used to retain potentially clinically relevant predictors. For immunological biomarkers, the more stringent lambda.1se criterion was applied to ensure robust incremental predictive value beyond established clinical factors. This strategy was based on the rationale that conventional clinical variables may carry broader clinical relevance, whereas novel immune-inflammatory biomarkers require a higher evidentiary threshold before being incorporated into the model.

LASSO regression identified eight predictors from the 17 routinely available clinical variables in the development cohort: sex, body mass index (BMI), mechanical thromboprophylaxis, mechanical ventilation, D-dimer, procalcitonin (PCT), C-reactive protein (CRP), and platelet-to-lymphocyte ratio (PLR) (Supplementary Figure S1a–b). In the exploratory immunological subgroup, interleukin-6 (IL-6) and absolute T-lymphocyte count were additionally selected from the 12 immune-inflammatory biomarkers (Supplementary Figure S1c–d).

As a sensitivity analysis, immune-inflammatory biomarker selection was repeated using the less stringent lambda.min criterion. Compared with the primary analysis using lambda.1se, lambda.min retained eight biomarkers (IL-2, IL-6, IL-17 A, TNF-α, absolute T-lymphocyte count, absolute B-lymphocyte count, absolute CD4 + T-cell count, and absolute NK-cell count) (Supplementary Figure S2). This finding indicates that the more conservative lambda.1se criterion selected a parsimonious subset of biomarkers while avoiding inclusion of additional variables that may increase the risk of overfitting in this relatively small exploratory subgroup.

### Model development and comparison

**Fig. 2:**
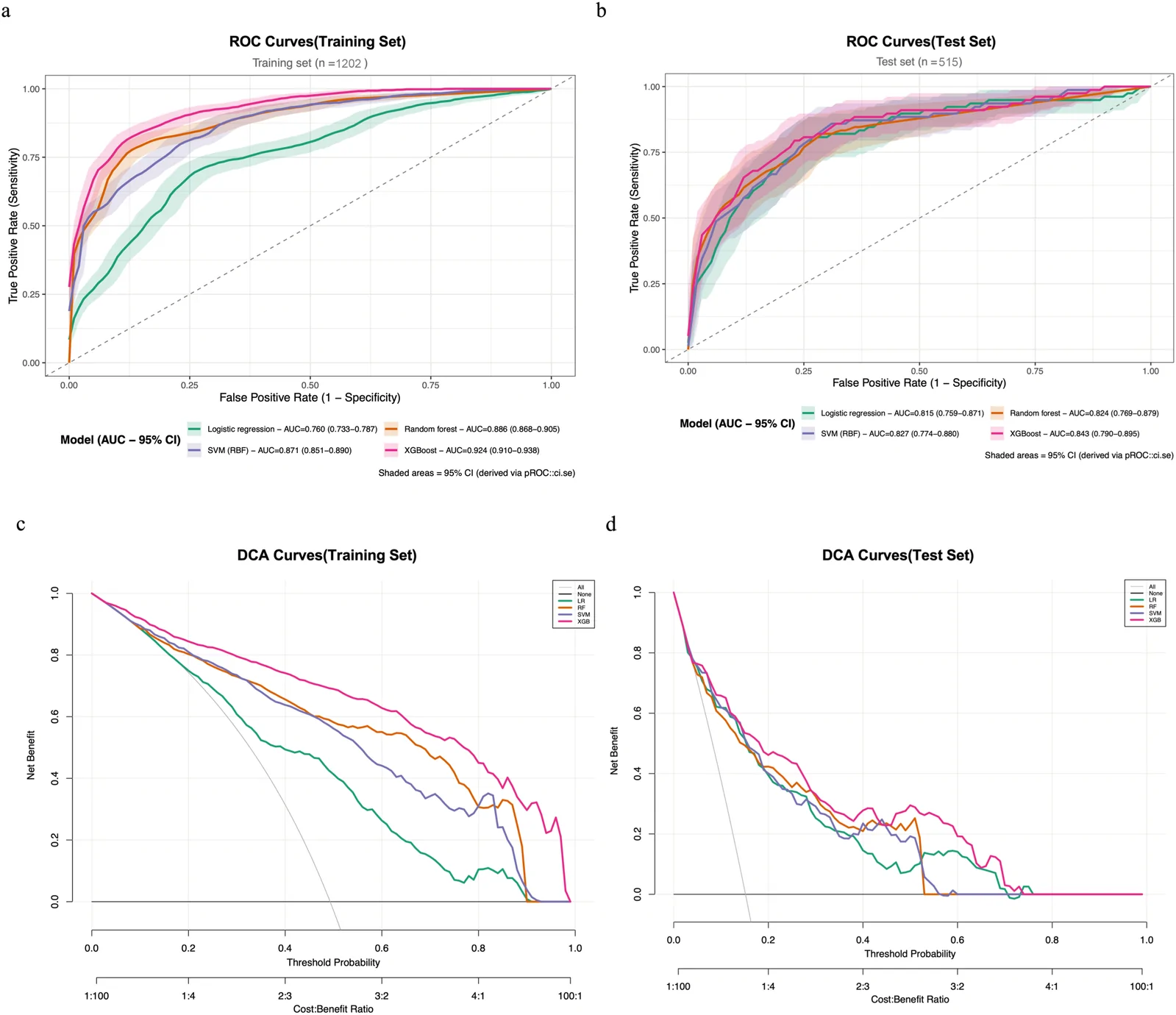
Discrimination and clinical utility of the prediction models. (**a–b**) Receiver operating characteristic (ROC) curves of logistic regression (LR), random forest (RF), support vector machine (SVM), and XGBoost models in the training and internal test cohorts. (**c–d**) Decision curve analysis (DCA) comparing the net benefit of the four models across different threshold probabilities

Four prediction models, including logistic regression, random forest, support vector machine, and extreme gradient boosting (XGBoost), were developed using 10-fold cross-validation. Model performance is summarized in Table 2, and receiver operating characteristic curves are shown in Fig. 2a–b. Logistic regression served as a conventional statistical benchmark, against which machine learning models were compared.

**Table 2:** Performance of machine learning models in the development cohort

Model	Dataset	AUC	Sensitivity (%)	Specificity (%)	Accuracy (%)	F1_Score	Brier_Score
LR	Training	0.76 (0.736–0.783)	64.6	77.4	71.1	68.8	0.199
RF	Training	0.886 (0.867–0.904)	84.1	74.9	79.5	80.2	0.145
SVM	Training	0.871 (0.851–0.892)	79.9	76.7	78.3	78.4	0.15
XGB	Training	0.924 (0.912–0.938)	83.8	85.6	84.7	84.4	0.117
LR	Test	0.815 (0.749–0.859)	66.7	81.7	79.4	49.5	0.174
RF	Test	0.824 (0.765–0.871)	70.5	78.4	77.2	48.5	0.157
SVM	Test	0.827 (0.774–0.878)	69.2	78.9	77.4	48.2	0.139
XGB	Test	0.843 (0.779–0.893)	67.9	84.4	81.9	53.3	0.126

Abbreviations: AUC, area under the receiver operating characteristic curve; CI, confidence interval; DVT, deep vein thrombosis; LR, logistic regression; RF, random forest; SVM, support vector machine; XGB, extreme gradient boosting. Model development and hyperparameter tuning were performed using 10-fold cross-validation in the training cohort. A fixed probability threshold of 0.5 was used to derive sensitivity, specificity, accuracy, and F1-score. AUC values are presented with 95% confidence intervals estimated using 1000 bootstrap iterations. Lower Brier scores indicate better calibration performance. Sensitivity, specificity, and accuracy are expressed as percentages. The test set refers to the internal validation cohort derived from the Suzhou development cohort (30% random split of the full dataset)

In the internal test cohort, the XGBoost model achieved an AUC of 0.843 (95% CI: 0.779 to 0.893), with sensitivity of 67.9% and specificity of 84.4%. Additional performance metrics included an F1-score of 0.533 and a Brier score of 0.126. Comparative performance across models is presented in Table 2, with XGBoost showing the highest discrimination among the evaluated methods. Decision curve analysis demonstrated net clinical benefit of the XGBoost model across a wide range of threshold probabilities in both the training and test cohorts (Fig. 2c–d). The XGBoost model was selected for external validation based on overall discrimination and decision curve performance.

### Sensitivity analysis

To examine the influence of D-dimer on model performance, a sensitivity analysis was conducted by excluding D-dimer from the candidate predictors. LASSO regression was repeated using the remaining 16 clinical variables, resulting in the selection of seven predictors (sex, body mass index, mechanical ventilation, mechanical thromboprophylaxis, procalcitonin, platelet-to-lymphocyte ratio, and C-reactive protein). All machine learning models were subsequently retrained. Overall model performance remained comparable with that of the primary analysis, with the XGBoost model achieving an AUC of 0.846 in the independent test cohort. Detailed results are presented in Supplementary Table S4. An additional sensitivity analysis was also performed by developing all machine learning models using all 17 candidate clinical predictors without LASSO-based feature selection. Predictive performance remained comparable to that of the primary LASSO-based models (Supplementary Table S5).

### Model interpretability

**Fig. 3:**
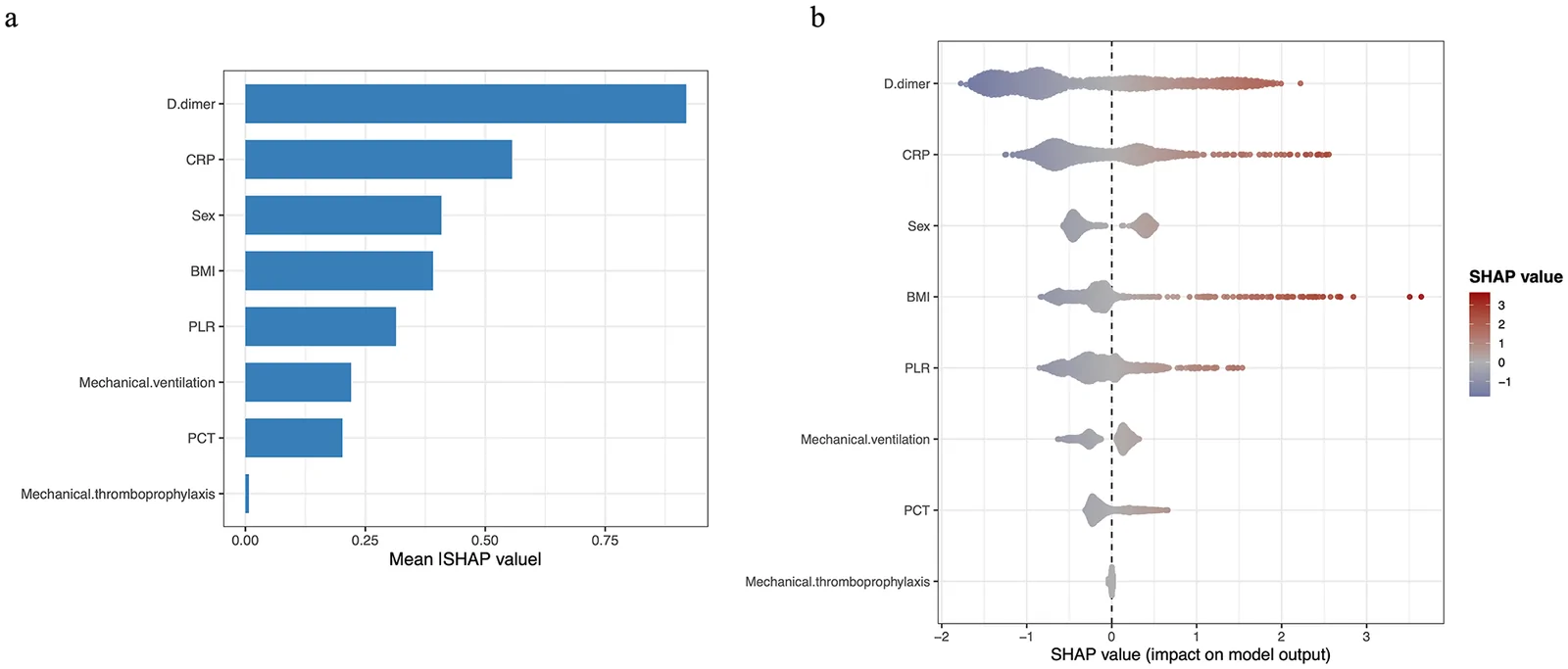
Model interpretability of the XGBoost algorithm using SHAP analysis. (**a**) Global feature importance ranked by mean absolute SHAP values. (**b**) SHAP summary plot showing the direction and magnitude of feature contributions across individual patients

SHapley Additive exPlanations (SHAP) were applied to the XGBoost model to improve model interpretability. D-dimer showed the highest global importance, followed by CRP, sex, BMI, PLR, mechanical ventilation, and PCT (Fig. 3a). SHAP summary plots further demonstrated that higher D-dimer and inflammatory marker levels generally contributed to an increased predicted risk of DVT (Fig. 3b). Representative SHAP dependence plots and individual force plots are provided in Supplementary Figure S3 to further illustrate feature-specific effects and patient-level prediction patterns.

### External validation

**Fig. 4:**
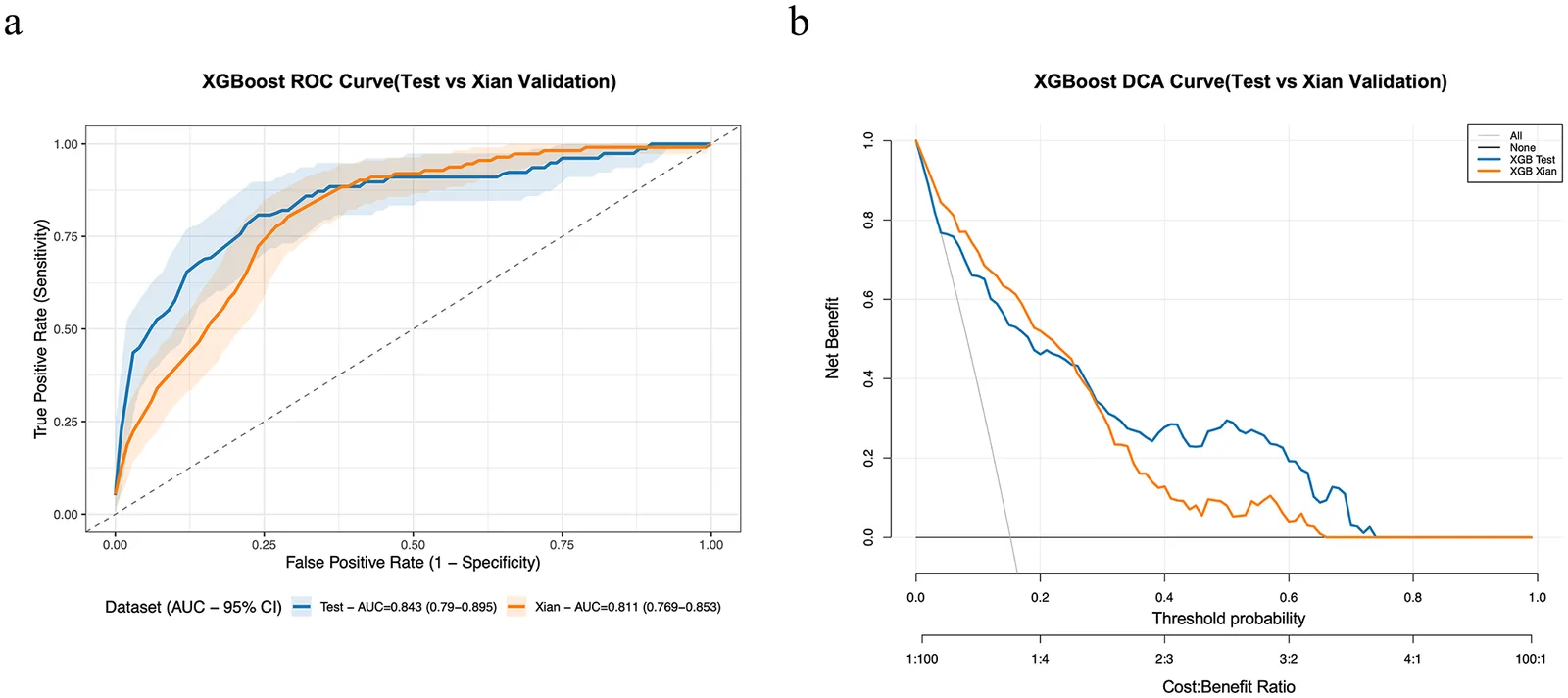
External validation of the XGBoost model. (**a**) Receiver operating characteristic (ROC) curves in the internal test cohort and external validation cohort. (**b**) Decision curve analysis (DCA) of the XGBoost model in the external validation cohort

The XGBoost model was externally validated in the independent Xi’an cohort (*n* = 533). The model achieved an AUC of 0.811 (95% CI: 0.769 to 0.853), with a sensitivity of 77.7%, specificity of 71.7%, accuracy of 73.0%, F1-score of 0.547, and Brier score of 0.158. Receiver operating characteristic and decision curve analyses demonstrated preserved discriminative ability and clinical utility in the external validation cohort (Fig. 4). Representative confusion matrices and predicted probability distributions are provided in Supplementary Figure S4.

### Model calibration

**Fig. 5:**
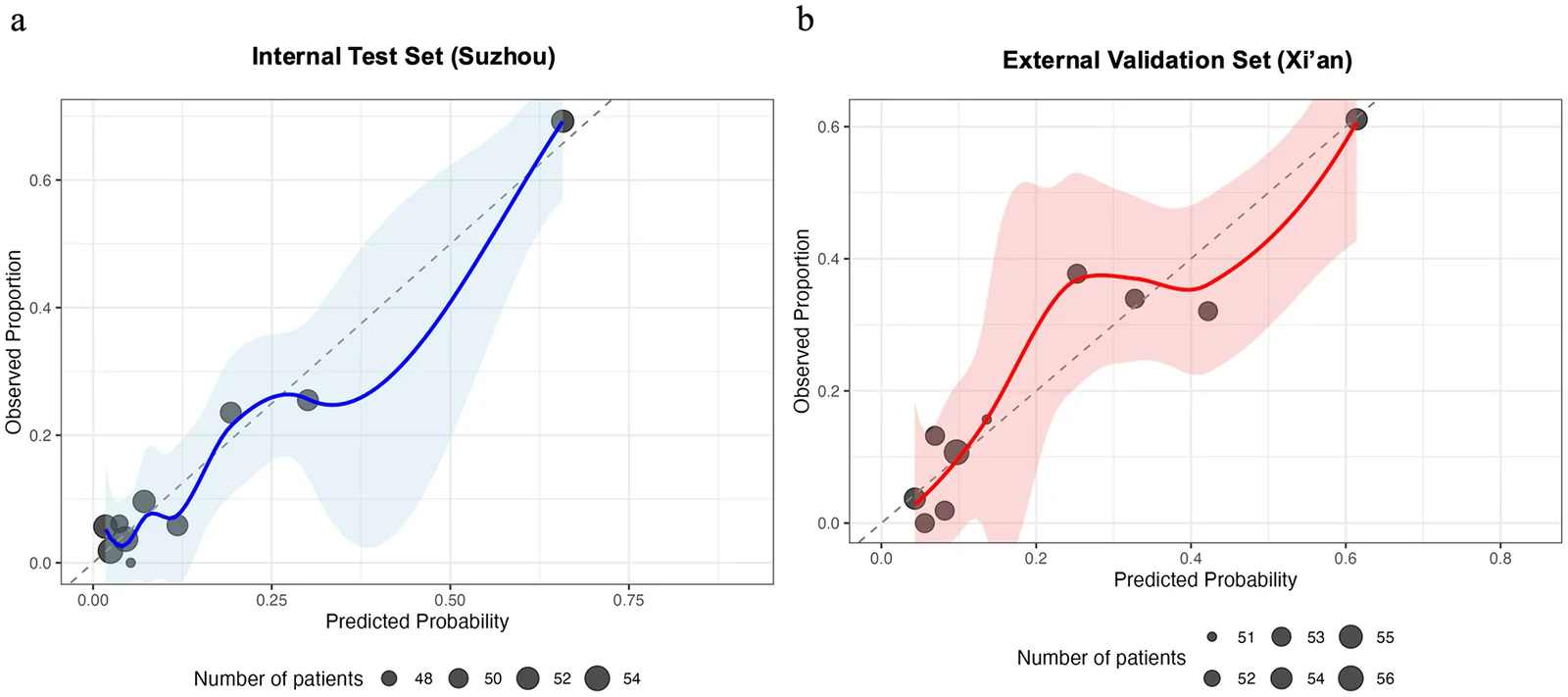
Calibration performance of the XGBoost model in the internal and external validation cohorts. (**a**) Internal validation cohort (Suzhou). (**b**) External validation cohort (Xi’an). The dashed diagonal line represents perfect calibration. Solid lines represent LOESS-smoothed calibration curves, and shaded areas indicate 95% confidence intervals. Predicted probabilities were grouped into deciles, with point sizes proportional to the number of patients in each decile. The model showed good agreement between predicted and observed DVT risk in both cohorts

Calibration of the XGBoost model was assessed in both the internal and external validation cohorts using calibration plots and quantitative metrics (Fig. 5; Table 3). In the internal validation cohort, the calibration plot demonstrated good agreement between predicted and observed DVT risk across the probability range (Fig. 5a), with a calibration intercept of -0.19 (95% CI: -0.35 to -0.05) and a calibration slope of 0.93 (95% CI: 0.79 to 1.08), indicating only minimal deviation from ideal calibration. The Spiegelhalter test showed no statistically significant evidence of miscalibration (*p =* 0.856).

**Table 3:** Calibration performance of the XGBoost model in the internal and external validation cohorts

Cohort	Calibration Intercept (95% CI)	Calibration Slope(95% CI)	Spiegelhalter *p*-value
Internal test set (Suzhou)	−0.19 (− 0.35 to − 0.05)	0.93 (0.79 to 1.08)	0.856
External validation set (Xi’an)	0.16 (− 0.02 to 0.34)	0.87 (0.71 to 1.04)	0.864

Calibration performance was evaluated in the internal validation cohort (Suzhou) and external validation cohort (Xi’an). Calibration intercepts and slopes are presented with 95% confidence intervals and were calculated using the original predicted probabilities. The Spiegelhalter goodness-of-fit test was used as a supplementary assessment of statistical evidence for miscalibration, with *p* > 0.05 indicating no statistically significant miscalibration

In the external validation cohort, similar calibration performance was observed (Fig. 5b), with a calibration intercept of 0.16 (95% CI: -0.02 to 0.34) and slope of 0.87 (95% CI: 0.71 to 1.04). The Spiegelhalter test remained non-significant (*p* = 0.864).

### Exploratory immunological subgroup analysis

In 191 patients with immune-inflammatory biomarker data, patients with DVT showed higher IL-6 levels and lower absolute T-lymphocyte counts compared with those without DVT (Supplementary Table S6).

**Fig. 6:**
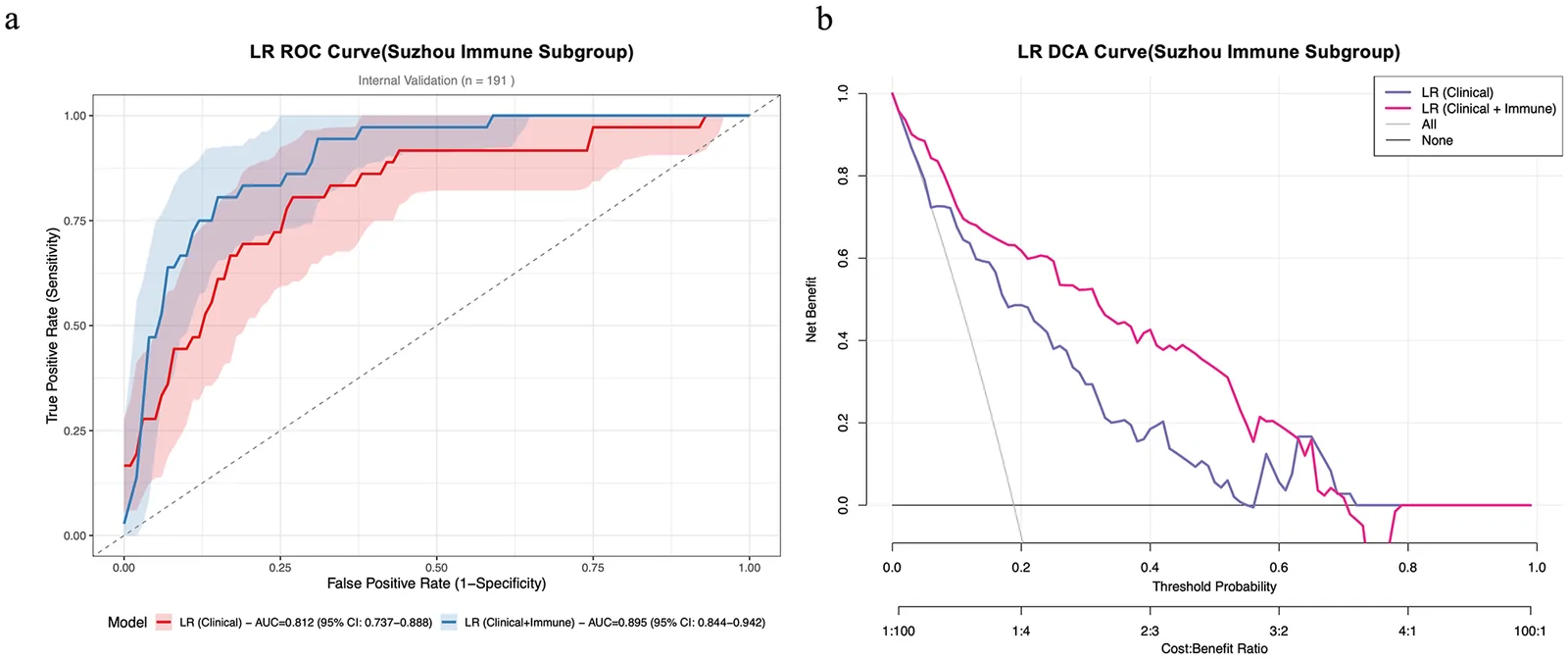
Exploratory subgroup analysis of immunological biomarkers. (**a**) Receiver operating characteristic (ROC) curves comparing the conventional clinical model and the combined clinical immunological model. (**b**) Decision curve analysis (DCA) comparing the net benefit of the two models

A weighted logistic regression model incorporating IL-6 and absolute T-lymphocyte count improved discrimination compared with the conventional clinical model, with the AUC increasing from 0.812 (95% CI: 0.737 to 0.888) to 0.895 (95% CI: 0.844 to 0.942) (Fig. 6a). The improvement in discrimination was statistically significant according to DeLong’s test (*p* = 0.008; Supplementary Table S7). Improvement was also observed in F1-score, sensitivity, and Brier score (Table 4). Because biomarker selection and model evaluation were performed within the same subgroup without independent validation, these findings should be interpreted as exploratory. Decision curve analysis indicated higher net benefit for the extended model across clinically relevant threshold probabilities (Fig. 6b).

**Table 4:** Comparison of conventional and combined clinical-immunological models

Model	AUC	Sensitivity (%)	Specificity (%)	Accuracy (%)	PPV (%)	NPV(%)		F1_Score	Brier_Score
LR (Clinical)	0.812(0.737–0.888)	69.4	80.0	78.0	44.6		91.9	0.543	0.173
LR(Clinical+Immune)	0.895 (0.844–0.942)	83.3	79.4	80.1	48.4		95.3	0.612	0.133

Sensitivity, specificity, accuracy, positive predictive value (PPV), and negative predictive value (NPV) are expressed as percentages

### Web-based calculator

**Fig. 7:**
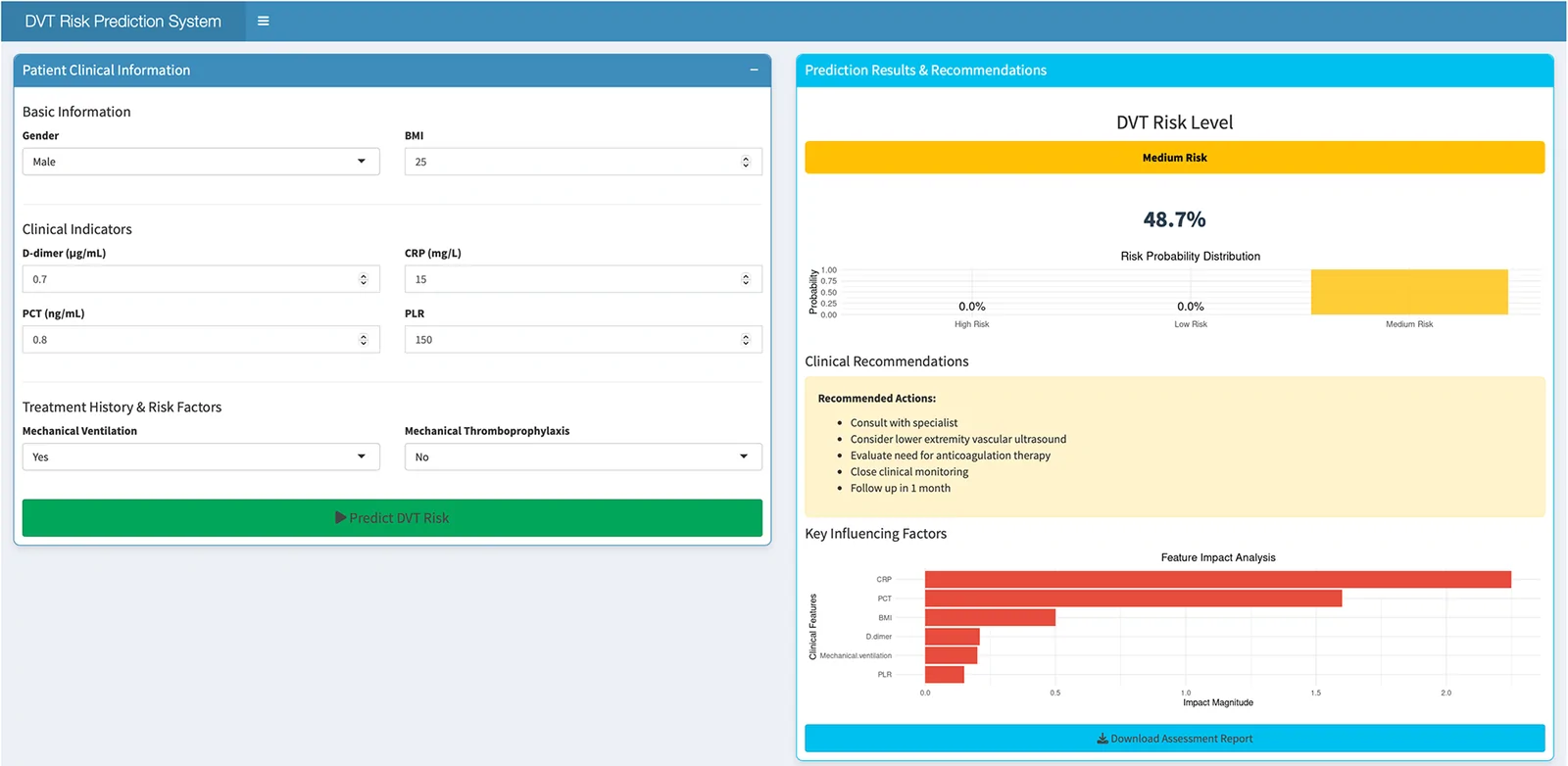
Web-based DVT risk calculator. Interface of the web-based calculator developed from the final XGBoost model for individualized prediction of DVT risk in elderly ICU patients using routinely available clinical variables

A web-based calculator based on the final XGBoost model was developed to facilitate clinical application (Fig. 7). The tool enables individualized risk estimation using routinely available clinical variables. The web calculator is available at: **[**https://clinical-application.shinyapps.io/dvt_shiny_app/**].**

## Discussion

In this multicenter retrospective study, we developed and externally validated an interpretable machine learning model for predicting deep vein thrombosis (DVT) in elderly intensive care unit (ICU) patients. The XGBoost model demonstrated stable discriminative performance in the external validation cohort, with an AUC of 0.811 (95% CI 0.769 to 0.853), while maintaining acceptable calibration across cohorts with different baseline event rates. Calibration performance was consistent in both the internal test set (intercept = -0.19 (95% CI: -0.35 to -0.05), slope = 0.93 (95% CI: 0.79 to 1.08), Spiegelhalter *p* = 0.856) and the external validation set (intercept = 0.16 (95% CI: -0.02 to 0.34), slope = 0.87 (95% CI: 0.71 to 1.04), Spiegelhalter *p* = 0.864), supporting good agreement between predicted and observed outcomes.

The exploratory subgroup analysis suggested that immune-inflammatory biomarkers, particularly IL-6 and absolute T-lymphocyte count, may add incremental predictive value beyond conventional clinical variables.

DVT remains a common and clinically significant complication in critically ill older adults and is associated with adverse outcomes and increased healthcare burden [1, 2]. Elderly ICU patients are at particularly high risk due to immobility, systemic inflammation, endothelial dysfunction, and multimorbidity [3]. However, existing clinical risk assessment tools were largely developed in broader populations and may not fully capture the complexity of critically ill elderly patients. Although direct calculation of Padua or Caprini scores was not feasible in this cohort due to incomplete data, the observed model performance appears comparable to or higher than previously reported ranges in ICU populations [23–25]. These comparisons remain indirect and should be interpreted cautiously.

Previous studies have applied machine learning methods to venous thromboembolism prediction in ICU settings [26–28]. Unlike previous prediction models developed in broader adult ICU populations, our study specifically focused on patients aged ≥ 75 years, a particularly vulnerable subgroup in whom accurate early risk stratification may be especially valuable. The findings support the applicability of interpretable machine learning approaches in a clinically high-risk but underrepresented population. The maintained performance in external validation suggests potential robustness across different ICU environments. In this study, logistic regression was used as a conventional baseline model, and machine learning methods demonstrated superior performance in terms of discrimination and overall predictive ability.

The predictors identified by the model are consistent with known mechanisms of thrombosis in critical illness. D-dimer showed the strongest contribution, consistent with its role in coagulation activation and fibrin turnover. Importantly, sensitivity analysis demonstrated that model performance remained largely unchanged after exclusion of D-dimer, indicating that the proposed model captures complementary predictive information from multiple routinely available clinical variables rather than relying solely on this established biomarker. Inflammatory markers such as CRP and PCT also contributed significantly, supporting the link between systemic inflammation and thrombosis risk [29, 30]. Mechanical ventilation and elevated BMI were associated with increased predicted risk, likely reflecting immobility, disease severity, and endothelial injury in critically ill patients [21].

SHAP-based interpretability analysis provided additional insight into feature contributions at both global and individual levels, supporting transparent interpretation of model predictions and enhancing clinical usability. This improves interpretability beyond traditional risk scores by enabling patient-specific risk explanation rather than population-level inference only.

The exploratory immunological subgroup analysis suggested that immune dysregulation may contribute to DVT risk. Patients with DVT had higher IL-6 levels and lower absolute T-lymphocyte counts, and incorporation of these biomarkers improved predictive performance. These findings align with emerging evidence on immunothrombosis, in which inflammatory and immune pathways interact with coagulation processes [29, 31]. Prior studies have also reported roles of cytokine activation, lymphocyte dysfunction, neutrophil extracellular traps, and platelet–leukocyte interactions in venous thrombosis [21, 30, 32]. However, given the limited sample size and exploratory design, these results should be considered hypothesis-generating.

From a clinical perspective, the model demonstrated potential utility across a range of decision thresholds in decision curve analysis, suggesting possible value in guiding targeted ultrasound screening and individualized thromboprophylaxis strategies [33, 34]. The associated web-based calculator enables bedside implementation using routinely available variables without requiring additional testing, supporting potential integration into clinical workflows.

This study has several strengths. The model was externally validated in an independent multicenter cohort, supporting generalizability. Calibration was systematically assessed using multiple complementary methods, including calibration plots, intercepts, slopes, and the Spiegelhalter test [35, 36]. In addition, SHAP analysis enhanced interpretability by enabling both global and patient-level explanation of predictions, improving clinical transparency.

Several limitations should be acknowledged. First, the retrospective design may introduce selection bias and limits causal inference. Second, external validation was restricted to two centers within a single healthcare system, and broader validation is required. Third, the immunological subgroup analysis was limited by the relatively small sample size and number of outcome events, increasing statistical uncertainty and the potential for model instability. Selection bias may also exist because only patients with available immune-inflammatory biomarker data were included. To further reduce the risk of overfitting, weighted logistic regression rather than machine learning algorithms was used in this subgroup; therefore, these findings should not be directly compared with those of the primary machine learning models. Moreover, sensitivity analysis using the less stringent LASSO *lambda.min* criterion retained substantially more immune-inflammatory biomarkers than the primary *lambda.1se* criterion, further supporting our decision to adopt the more conservative feature-selection strategy in this relatively small exploratory subgroup. Although multicollinearity was assessed using VIF analysis, nonlinear relationships between continuous predictors and the logit were not formally evaluated using methods such as the Box–Tidwell test. Because the LR models served primarily as baseline comparators rather than the primary predictive models, this is unlikely to materially affect the main findings but should be acknowledged as a methodological limitation.

Fourth, because biomarker selection and model evaluation were performed within the same immunological subgroup, some degree of optimism bias cannot be excluded despite the statistically significant improvement demonstrated by DeLong’s test. Accordingly, the incremental predictive value of IL-6 and absolute T-lymphocyte count should be considered exploratory and requires validation in larger independent prospective cohorts before clinical implementation.

Fifth, temporal changes in clinical variables during ICU stay were not captured [37]. Furthermore, certain clinically relevant physical examination findings, such as calf circumference or unilateral calf swelling, were not routinely documented in the retrospective electronic medical records. As our model was designed for early prediction using variables collected within the first 24 h after ICU admission, these physical examination findings could not be incorporated into the current analysis. Future prospective studies with standardized assessment of these clinical signs may further improve predictive performance.

Finally, bleeding risk was not assessed, although it is essential when balancing thrombosis prevention and anticoagulation strategies in critically ill patients [38].

Future work should focus on prospective multicenter validation, real-world clinical impact assessment, and incorporation of longitudinal data to enable dynamic prediction modeling. Larger prospective multicenter studies are needed to further evaluate the role of immunothrombosis in DVT development [29].

## Conclusion

We developed and externally validated an interpretable machine learning model based on XGBoost for early prediction of deep vein thrombosis in elderly ICU patients. The model demonstrated good discrimination and calibration in an independent external cohort, supporting its robustness across different clinical settings. The integration of SHAP-based interpretability enables transparent risk explanation at both the population and individual levels, facilitating clinical interpretability beyond conventional risk scores. The web-based application further provides a practical decision support tool for individualized bedside risk estimation using routinely available clinical variables. Exploratory analysis suggested that immune-inflammatory biomarkers may provide additional predictive value and are associated with immunothrombosis-related mechanisms. Further prospective validation and real-world implementation studies are required to evaluate its clinical impact and utility in routine decision-making.

## Supplementary Information

Below is the link to the electronic supplementary material.

Supplementary Material 1: Supplementary Figure S1. Variable selection using the LASSO regression method. Supplementary Figure S2. Sensitivity analysis of immune-inflammatory biomarker selection using the LASSO regression method with the lambda.min criterion. Supplementary Figure S3. Additional SHAP visualizations of the XGBoost model. Supplementary Figure S4. Additional external validation results of the XGBoost model. Supplementary Table S1. Missing data rates of candidate predictors before imputation. Supplementary Table S2. Events-per-variable (EPV) calculation for the baseline logistic regression models. Supplementary Table S3. Variance inflation factor (VIF) analysis for predictors included in the baseline logistic regression models. Supplementary Table S4. Sensitivity analysis of machine learning models after exclusion of D-dimer from the candidate predictor set. Supplementary Table S5. Sensitivity analysis of machine learning models developed using all candidate clinical predictors without LASSO feature selection. Supplementary Table S6. Baseline immune-inflammatory biomarker profiles in the exploratory immunological subgroup (n = 191). Supplementary Table S7. Comparison of discriminative performance between the conventional clinical model and the combined clinical–immunological model using DeLong’s test.

## Data Availability

The datasets analyzed during the current study and the corresponding code are available from the corresponding author on reasonable request.

## Abbreviations

AUC: Area under the curve
BMI: Body mass index
CI: Confidence interval
CRP: C-reactive protein
DCA: Decision curve analysis
DVT: Deep vein thrombosis
ICU: Intensive care unit
LASSO: Least absolute shrinkage and selection operator
LR: Logistic regression
ML: Machine learning
MICE: Multiple imputation by chained equations
NLR: Neutrophil-to-lymphocyte ratio
NPV: Negative predictive value
PCT: Procalcitonin
PE: Pulmonary embolism
PLR: Platelet-to-lymphocyte ratio
PPV: Positive predictive value
RF: Random forest
ROC: Receiver operating characteristic
SHAP: SHapley additive exPlanations
SMD: Standardized mean difference
SVM: Support vector machine
TNF-α: Tumor necrosis factor-alpha
VTE: Venous thromboembolism
WBC: White blood cell
XGB/XGBoost: Extreme gradient boosting
