# Supplementary material for: Interpretable machine learning model for early prediction of deep vein thrombosis in elderly ICU patients: multicenter development and external validation
Source: BMC Med Inform Decis Mak. 2026 Aug 8;26:415. doi: 10.1186/s12911-026-03747-z (PMC13673899; doi:10.1186/s12911-026-03747-z)
Supplement: Supplementary file 1 [file 12911_2026_3747_MOESM1_ESM.docx]

**Supplementary Materials**

**Title:**

Interpretable Machine Learning Model for Early Prediction of Deep Vein Thrombosis in Elderly ICU Patients: Multicenter Development and External Validation

**Manuscript ID:**

bc8c1202-5cce-42e7-981e-03f1ee22066c

 **Supplementary Figure S1**. Variable selection using the LASSO regression method. (a–b) Variable selection process for conventional clinical predictors, showing the LASSO coefficient profiles and the optimal penalty parameter (λ) identified by minimum cross-validation error (lambda.min), which retained eight key predictors. (c–d) Variable selection process for immunological biomarkers, displaying the LASSO coefficient profiles and the optimal λ determined by the one-standard-error rule (lambda.1se), which retained two immunological predictors.

**Supplementary Figure S2.** Sensitivity analysis of immune-inflammatory biomarker selection using the LASSO regression method with the lambda.min criterion. (a–b) Variable selection process for immune-inflammatory biomarkers using the less stringent lambda.min criterion, showing the LASSO coefficient profiles and the optimal penalty parameter (λ) identified by minimum cross-validation error (lambda.min), which retained eight immune-inflammatory biomarkers (IL-2, IL-6, IL-17A, TNF-α, absolute T-lymphocyte count, absolute B-lymphocyte count, absolute CD4⁺ T-cell count, and absolute NK-cell count). Compared with the primary lambda.1se criterion, this approach selected a substantially larger number of predictors, supporting the use of the more conservative lambda.1se criterion in the primary analysis to reduce the risk of overfitting.

 **Supplementary Figure S3**. Additional SHAP visualizations of the XGBoost model. (a) SHAP dependence plots for representative predictors (e.g., D-dimer and CRP), illustrating the relationships between predictor values and SHAP contributions. (b) SHAP force plots for two representative patients, demonstrating individualized prediction patterns.

 **Supplementary Figure S4**. Additional external validation results of the XGBoost model. (a) Confusion matrix of model classification performance in the external validation cohort. (b) Distribution of predicted probabilities in patients with and without DVT.

**Supplementary Table S1. Number and percentage of missing values for candidate predictors before imputation**

| **Conventional clinical predictors** | **Missing n (%)** | **Immunological biomarkers** | **Missing n (%)** |
| --- | --- | --- | --- |
| Age | 0 (0.0%) | Absolute T lymphocyte count | 5 (2.6%) |
| Sex | 0 (0.0%) | Absolute NK cell count | 7 (3.7%) |
| Body mass index (BMI) | 18 (1.0%) | Absolute B lymphocyte count | 4 (2.1%) |
| APACHE II score | 13 (0.8%) | Absolute CD8+ T cell count | 8 (4.2%) |
| Diabetes | 0 (0.0%) | Absolute CD4+ T cell count | 6 (3.1%) |
| Hypertension | 2 (0.1%) | Interleukin-6 (IL-6) | 9 (4.7%) |
| Malignancy | 0 (0.0%) | Interleukin-17A (IL-17A) | 10 (5.2%) |
| Pharmacological thromboprophylaxis | 7 (0.4%) | Interleukin-1β (IL-1β) | 3 (1.6%) |
| Mechanical thromboprophylaxis | 0 (0.0%) | Interleukin-10 (IL-10) | 5 (2.6%) |
| Orthopedic surgery | 22(1.3%) | Interleukin-2 (IL-2) | 6 (3.1%) |
| Mechanical ventilation | 0 (0.0%) | Tumor necrosis factor-alpha (TNF-α) | 4 (2.1%) |
| D-dimer | 46 (2.7%) | Interferon-gamma (IFN-γ) | 8 (4.2%) |
| Neutrophil-to-lymphocyte ratio (NLR) | 28 (1.6%) |  |  |
| Platelet-to-lymphocyte ratio (PLR) | 35 (2.0%) |  |  |
| White blood cell count (WBC) | 11 (0.6%) |  |  |
| Procalcitonin (PCT) | 38 (2.2%) |  |  |
| C-reactive protein (CRP) | 24 (1.4%) |  |  |

**Abbreviations:** BMI, body mass index; APACHE II, Acute Physiology and Chronic Health Evaluation II; PCT, procalcitonin; CRP, C-reactive protein; NLR, neutrophil-to-lymphocyte ratio; PLR, platelet-to-lymphocyte ratio; IL, interleukin; TNF-α, tumor necrosis factor-α; IFN-γ, interferon-γ; NK, natural killer.

Missing continuous variables were imputed using multiple imputation by chained equations (MICE), whereas missing categorical variables were imputed using mode imputation before model development.

**Supplementary Table S2. Events-per-variable (EPV) calculation for the baseline logistic regression models.**

| **Model** | **Sample size(n)** | **Outcome events, n** | **Predictors, n** | **EPV** |
| --- | --- | --- | --- | --- |
| Clinical LR | 1717 | 261 | 8 | 32.6 |
| Immune LR | 191 | 36 | 2 | 18 |

**Abbreviations:** EPV, events per variable; LR, logistic regression. EPV was calculated as the ratio of outcome events to the number of predictors included in each baseline logistic regression model. An EPV value ≥10 is generally considered sufficient to reduce the risk of overfitting and ensure stable coefficient estimation in logistic regression models.

**Supplementary Table S3. Variance inflation factor (VIF) analysis for predictors included in the baseline logistic regression models**

| **Model** | **Predictor** | **VIF** |
| --- | --- | --- |
| Clinical LR | Sex | 1.03 |
|  | BMI | 1.04 |
|  | Mechanical ventilation | 1.11 |
|  | Mechanical thromboprophylaxis | 1.13 |
|  | D-dimer | 1.18 |
|  | PCT | 1.21 |
|  | CRP | 1.19 |
|  | PLR | 1.31 |
| Immune LR | IL-6 | 1.20 |
|  | Absolute T lymphocyte count | 1.08 |

**Abbreviations:** VIF, variance inflation factor; LR, logistic regression; BMI, body mass index; PCT, procalcitonin; CRP, C-reactive protein; PLR, platelet-to-lymphocyte ratio; IL-6, interleukin-6. VIF was calculated to assess multicollinearity among predictors included in the baseline logistic regression models. A VIF value <5 was considered indicative of the absence of substantial multicollinearity. Where complete collinearity prevented VIF estimation, collinear variables were excluded from the VIF analysis.

**Supplementary Table S4. Sensitivity analysis of machine learning models after exclusion of D-dimer from the candidate predictor set**

| **Model** | **Dataset** | **AUC** | **Sensitivity**  **(%)** | **Specificity**  **(%)** | **Accuracy**  **(%)** | **F1_Score** | **Brier_Score** |
| --- | --- | --- | --- | --- | --- | --- | --- |
| LR | Training | 0.832 (0.781-0.883) | 76.9 | 77.3 | 77.2 | 50.6 | 0.164 |
| RF | Training | 0.857（0.840-0.909） | 79.5 | 86.5 | 84.2 | 66.3 | 0.129 |
| SVM | Training | 0.895 (0.877-0.913) | 78.4 | 85.1 | 82.5 | 80.1 | 0.128 |
| XGB | Training | 0.904 (0.891-0.916) | 86.5 | 90.2 | 88.4 | 88 | 0.091 |
| LR | Test | 0.799 (0.774-0.824) | 69.8 | 76.4 | 73.2 | 71.9 | 0.182 |
| RF | Test | 0.841 (0.787-0.895) | 71.8 | 83.2 | 81.7 | 58 | 0.121 |
| SVM | Test | 0.831 (0.776-0.885) | 67.9 | 83.1 | 80.8 | 54.1 | 0.132 |
| XGB | Test | 0.846 (0.792-0.899) | 75.4 | 88.5 | 85.4 | 68.9 | 0.101 |

Abbreviations: AUC, area under the receiver operating characteristic curve; CI, confidence interval; DVT, deep vein thrombosis; LR, logistic regression; RF, random forest; SVM, support vector machine; XGB, extreme gradient boosting. D-dimer was removed before feature selection. LASSO regression was repeated using the remaining candidate clinical variables, and all machine learning models were retrained using the same modeling strategy as the primary analysis.

**Supplementary Table S5. Sensitivity analysis of machine learning models developed using all candidate clinical predictors without LASSO feature selection**

| **Model** | **Dataset** | **AUC** | **Sensitivity**  **(%)** | **Specificity**  **(%)** | **Accuracy**  **(%)** | **F1_Score** | **Brier_Score** |
| --- | --- | --- | --- | --- | --- | --- | --- |
| LR | Training | 0.821 (0.797-0.844) | 73.4 | 76.4 | 74.9 | 74.2 | 0.172 |
| RF | Training | 0.857 (0.840-0.909) | 79.5 | 83.2 | 81.7 | 66.3 | 0.129 |
| SVM | Training | 0.895 (0.877-0.913) | 78.4 | 85.1 | 82.5 | 80.1 | 0.128 |
| XGB | Training | 0.953 (0.942-0.964) | 86.5 | 90.2 | 88.4 | 88 | 0.091 |
| LR | Test | 0.805 (0.775-0.835) | 69.8 | 77.5 | 74.8 | 67.3 | 0.181 |
| RF | Test | 0.841 (0.787-0.895) | 71.8 | 86.5 | 84.2 | 58 | 0.121 |
| SVM | Test | 0.831 (0.776-0.885) | 67.9 | 83.1 | 80.8 | 54.1 | 0.132 |
| XGB | Test | 0.860 (0.813-0.907) | 75.4 | 88.5 | 85.4 | 68.9 | 0.101 |

Abbreviations: AUC, area under the receiver operating characteristic curve; CI, confidence interval; DVT, deep vein thrombosis; LR, logistic regression; RF, random forest; SVM, support vector machine; XGB, extreme gradient boosting. As a sensitivity analysis, four prediction models (logistic regression, random forest, support vector machine, and extreme gradient boosting [XGBoost]) were developed using all 17 candidate clinical predictors without prior LASSO-based variable selection. Model performance was evaluated in the training and internal test cohorts using the area under the receiver operating characteristic curve (AUC), sensitivity, specificity, accuracy, F1-score, and Brier score. AUC values are presented with 95% confidence intervals estimated using 1,000 bootstrap resamples. These results were used to assess whether variable selection by LASSO materially affected predictive performance.

**Supplementary Table S6. Baseline immune-inflammatory biomarker profiles in the exploratory immunological subgroup (n = 191)**

| **Characteristics** | **DVT**  **（n=36）** | **No-DVT**  **（n=155）** | **P^2^ value** |
| --- | --- | --- | --- |
| IL-1β, pg/mL | 11.37 (8.30, 16.30) | 11.30 (7.30, 12.60) | 0.8 |
| IL-2, pg/mL | 5.65 (3.24, 12.15) | 5.90 (3.40, 12.40) | 0.9 |
| IL-6, pg/mL | 19.30 (12.35, 27.30) | 11.60 (8.30, 16.90) | <0.001 |
| IL-10, pg/mL | 10.70 (4.50, 15.70) | 9.90 (6.34, 14.50) | 0.8 |
| IL-17A, pg/mL | 17.85 (12.10, 29.60) | 14.20 (9.30, 21.30) | 0.012 |
| TNF-α, pg/mL | 7.35 (3.50, 15.50) | 4.60 (3.10, 9.20) | 0.068 |
| IFN-γ, pg/mL | 4.80 (3.00, 7.17) | 4.75 (3.40, 8.26) | 0.7 |
| Absolute T lymphocyte count, cells/µL | 564.00 (463.00, 773.00) | 799.00 (657.00, 999.00) | <0.001 |
| Absolute B lymphocyte count, cells/µL | 239.50 (103.50, 404.00) | 289.00 (215.00, 369.00) | 0.4 |
| Absolute CD8+ T cell count, cells/µL | 235.00 (154.50, 326.00) | 269.00 (218.00, 389.00) | 0.038 |
| Absolute CD4+ T cell count, cells/µL | 328.50 (202.00, 416.50) | 287.00 (216.00, 367.00) | 0.7 |
| Absolute NK cell count, cells/µL | 154.00 (105.00, 245.00) | 213.00 (167.00, 298.00) | 0.002 |

Continuous variables are presented as median [interquartile range]. Between-group comparisons (DVT vs non-DVT) were performed using the Wilcoxon rank-sum test. P-values are shown and a two-sided test was applied.

**Supplementary Table S7. Comparison of discriminative performance between the conventional clinical model and the combined clinical–immunological model using DeLong’s test**

| **Comparison** | | **ΔAUC** | **DeLong *p*** |
| --- | --- | --- | --- |
|  | Clinical vs Clinical+Immune | 0.083 | 0.008 |

Abbreviations: AUC, area under the receiver operating characteristic curve.

ΔAUC represents the absolute difference in AUC between the combined clinical–immunological model and the conventional clinical model. Statistical significance of the difference between the two correlated ROC curves was assessed using DeLong’s test.
